# Trends in Development of Novel Machine Learning Methods for the Identification of Gliomas in Datasets That Include Non-Glioma Images: A Systematic Review

**DOI:** 10.3389/fonc.2021.788819

**Published:** 2021-12-23

**Authors:** Harry Subramanian, Rahul Dey, Waverly Rose Brim, Niklas Tillmanns, Gabriel Cassinelli Petersen, Alexandria Brackett, Amit Mahajan, Michele Johnson, Ajay Malhotra, Mariam Aboian

**Affiliations:** ^1^ Department of Radiology and Biomedical Imaging, Yale School of Medicine, New Haven, CT, United States; ^2^ Harvey Cushing/John Hay Whitney Medical Library, Yale School of Medicine, New Haven, CT, United States

**Keywords:** artificial intelligence, bias, brain tumor, diagnostic imaging, glioma, machine learning, Magnetic Resonance Imaging, segmentation

## Abstract

**Purpose:**

Machine learning has been applied to the diagnostic imaging of gliomas to augment classification, prognostication, segmentation, and treatment planning. A systematic literature review was performed to identify how machine learning has been applied to identify gliomas in datasets which include non-glioma images thereby simulating normal clinical practice.

**Materials and Methods:**

Four databases were searched by a medical librarian and confirmed by a second librarian for all articles published prior to February 1, 2021: Ovid Embase, Ovid MEDLINE, Cochrane trials (CENTRAL), and Web of Science-Core Collection. The search strategy included both keywords and controlled vocabulary combining the terms for: artificial intelligence, machine learning, deep learning, radiomics, magnetic resonance imaging, glioma, as well as related terms. The review was conducted in stepwise fashion with abstract screening, full text screening, and data extraction. Quality of reporting was assessed using TRIPOD criteria.

**Results:**

A total of 11,727 candidate articles were identified, of which 12 articles were included in the final analysis. Studies investigated the differentiation of normal from abnormal images in datasets which include gliomas (7 articles) and the differentiation of glioma images from non-glioma or normal images (5 articles). Single institution datasets were most common (5 articles) followed by BRATS (3 articles). The median sample size was 280 patients. Algorithm testing strategies consisted of five-fold cross validation (5 articles), and the use of exclusive sets of images within the same dataset for training and for testing (7 articles). Neural networks were the most common type of algorithm (10 articles). The accuracy of algorithms ranged from 0.75 to 1.00 (median 0.96, 10 articles). Quality of reporting assessment utilizing TRIPOD criteria yielded a mean individual TRIPOD ratio of 0.50 (standard deviation 0.14, range 0.37 to 0.85).

**Conclusion:**

Systematic review investigating the identification of gliomas in datasets which include non-glioma images demonstrated multiple limitations hindering the application of these algorithms to clinical practice. These included limited datasets, a lack of generalizable algorithm training and testing strategies, and poor quality of reporting. The development of more robust and heterogeneous datasets is needed for algorithm development. Future studies would benefit from using external datasets for algorithm testing as well as placing increased attention on quality of reporting standards.

**Systematic Review Registration:**

www.crd.york.ac.uk/prospero/display_record.php?ID=CRD42020209938, International Prospective Register of Systematic Reviews (PROSPERO 2020 CRD42020209938).

## Introduction

As the healthcare needs of the population increase and the volume of imaging grows, there is a critical need for computer assisted models to provide support to radiologists in routine clinical practice. Brain tumors, and specifically gliomas, are of particular interest to neuro-oncologists and radiologists. Machine learning research in neuro-oncology has become increasingly popular as sufficient computing power and large datasets have come to be more available to researchers. Machine learning refers to a subset of artificial intelligence consisting of algorithms that analyze data without explicit programming (1, 2). Deep learning is a subtype of machine learning that utilizes neural networks, which refer to algorithm models composed of neurons represented by nodes and interconnections between nodes (3). Machine learning has been applied to the diagnostic imaging of gliomas to augment classification, prognostication, segmentation, and treatment planning (4). Algorithms which can differentiate gliomas from other entities such as normal examinations, stroke, or demyelinating disease remain in the early stages of development. Until now, most studies have focused on brain tumor segmentation accuracy, and provide segmentation algorithms which are developed on datasets containing only glioma images. The identification of gliomas in a heterogeneous group of images is a critical function but less well studied. In clinical practice, most studies contain normal images or other non-oncologic pathology. Algorithms developed on datasets containing only glioma images are unlikely to be generalizable to clinical practice. Therefore, in this study we investigate how machine learning has been applied to the identification of gliomas in datasets which contain non-glioma images. A systematic review was performed to assess the existing body of literature and identify the most optimal targets for future research.

## Materials and Methods

**Figure 1:**
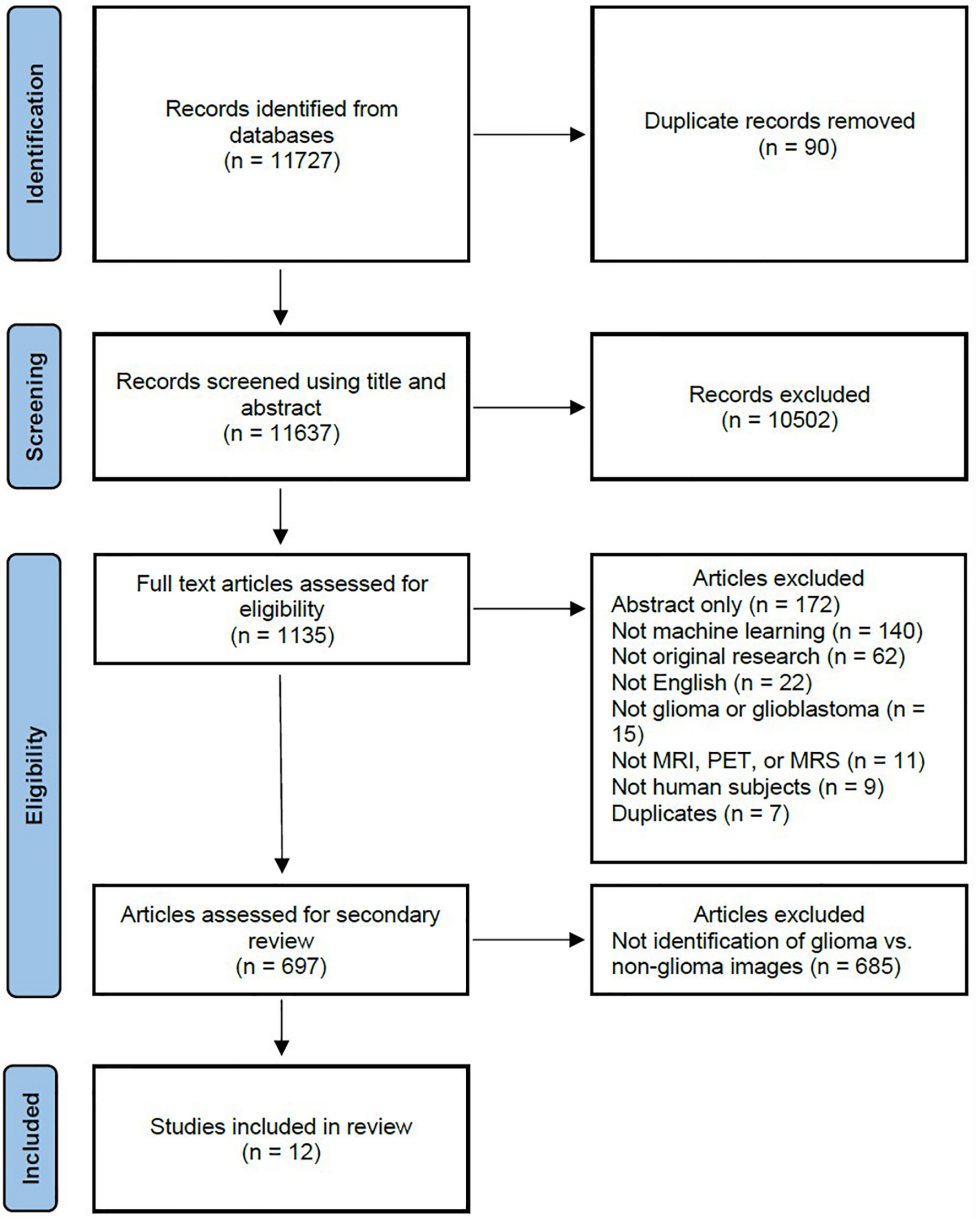
PRISMA flow diagram depicting the systematic review search strategy. (MRI, magnetic resonance imaging; MRS, magnetic resonance spectroscopy; PET, positron emission tomography.).

A systematic literature review was performed to identify how machine learning has been applied to identify gliomas in datasets which include non-glioma images, thereby simulating normal clinical practice. The study was registered with the International Prospective Register of Systematic Reviews (PROSPERO, CRD42020209938) and conducted in concordance with preferred reporting items for systematic review and meta-analysis protocols (PRISMA-P) guidelines (5). The primary literature search is summarized in the PRISMA flow diagram in **Figure 1**, and involved a query of four databases to identify all published articles investigating machine learning and gliomas. The queried databases were Ovid Embase, Ovid MEDLINE, Cochrane trials (CENTRAL), and Web of Science-Core Collection. The initial search included articles published prior to September 1, 2020, and a second search was performed to identify articles published between September 1, 2020 and February 1, 2021. The search strategy included both keywords and controlled vocabulary combining the terms for: artificial intelligence, machine learning, deep learning, radiomics, magnetic resonance imaging, glioma, as well as related terms. The search strategy and syntax are demonstrated in **Supplementary Figure S1**. The search was executed by a medical librarian and reviewed by a second institutional librarian.

Screening of the articles was performed by two independent reviewers (H.S. and M.A.), which includes one board certified neuroradiologist (M.A.), utilizing Covidence (Covidence systematic review software, Veritas Health Innovation, Melbourne, Australia. Available at www.covidence.org). Articles were initially screened by title and abstract, after which the remaining articles were screened by full text. To meet inclusion criteria, the articles were required to be original research, investigate machine learning, investigate gliomas in human subjects, be published in the English language, and utilize imaging with either MRI, MRS, or PET. Further screening was then performed to identify articles which investigated the identification of gliomas in datasets including non-glioma images. Each reviewer screened each article independently and disagreement was resolved by discussion.

Data extraction was performed by two independent reviewers (H.S. and R.D.). Each reviewer extracted the whole data independently and disagreement was resolved by discussion. Major data points included the study objective, dataset, number of patients and images, machine learning algorithm training and testing strategy, and magnetic resonance imaging (MRI) sequences. Quantitative data was also collected where available, including accuracy, sensitivity, specificity, area under the receiver operating characteristic curve (AUC) and Dice coefficient. When multiple algorithms were evaluated in a study, the best performing algorithm was reported.

Risk of bias assessment was performed using Transparent Reporting of a Multivariable Prediction Model for Individual Prognosis or Diagnosis (TRIPOD) guidelines (6). The TRIOPD checklist contains 22 primary features as well as multiple subitems, resulting in a total of 37 features. A TRIPOD score was created using 1 possible point for each subitem. Adherence to a subitem was given 1 point, while non-adherence was scored as 0 points. Features not assessed in an article due to the nature of the study were deemed as not applicable and excluded from analysis. The primary features are title (1 point), abstract (1 point), introduction - background and objectives (2 points), methods - source of data (2 points), methods - participants (3 points), methods - outcome (2 points), methods - predictors (2 points), methods - sample data (1 point), methods - missing data (1 point), methods - statistical analysis (5 points), methods - risk groups (1 point), methods - development and validation (1 point), results - participants (3 points), results - model development (2 points), results - model specification (2 points), results - model performance (1 point), results - model updating (1 point), discussion - limitations (1 point), discussion - interpretation (2 points), discussion - implications (1 point), supplementary information (1 point) and funding (1 point). The individual TRIPOD ratio was calculated for each article as the ratio of the TRIPOD score to the maximum possible points calculated from the included features. The TRIPOD adherence ratio for each feature was calculated as the ratio of the total points for a specific feature to the total possible points from all of the articles assessing that feature.

Descriptive statistics were calculated and visualized using GraphPad Prism version 9.1.2 for Windows, GraphPad Software, San Diego, California USA, www.graphpad.com.

## Results

The primary literature search returned 11,727 candidate articles, of which 90 duplicates were removed. The remaining 11,637 articles were screened using title and abstract, of which 10,502 articles that did not involve neuro-oncology were excluded. The full text of the remaining 1,135 articles was reviewed, of which 438 articles were excluded. The 438 excluded articles consisted of 172 conference abstracts, 140 articles not utilizing machine learning, 62 not representing original research, 22 not published in the English language, 15 not investigating gliomas, 11 not utilizing MRI, magnetic resonance spectroscopy (MRS), or positron emission tomography (PET) imaging, 9 not utilizing human subjects, and 7 duplicate articles. The remaining 697 articles underwent further review, of which 685 articles were excluded and 12 articles (7–18) investigating the use of machine learning to identify gliomas in datasets which include non-glioma images were identified for inclusion in the final analysis.

**Figure 2:**
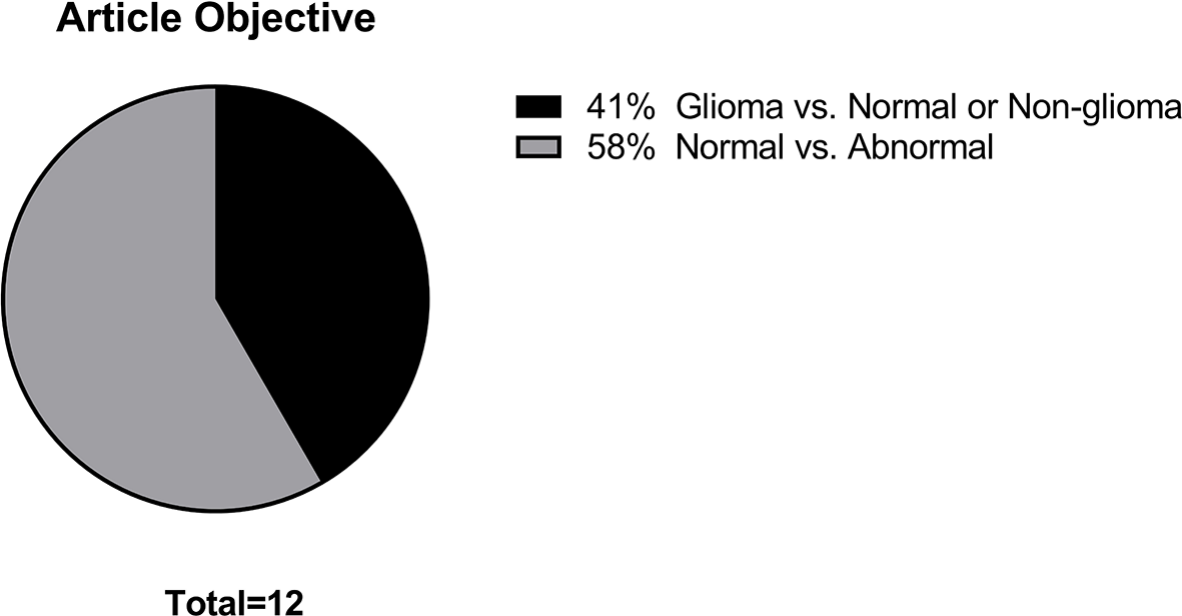
Distribution of article objectives.

**Figure 3:**
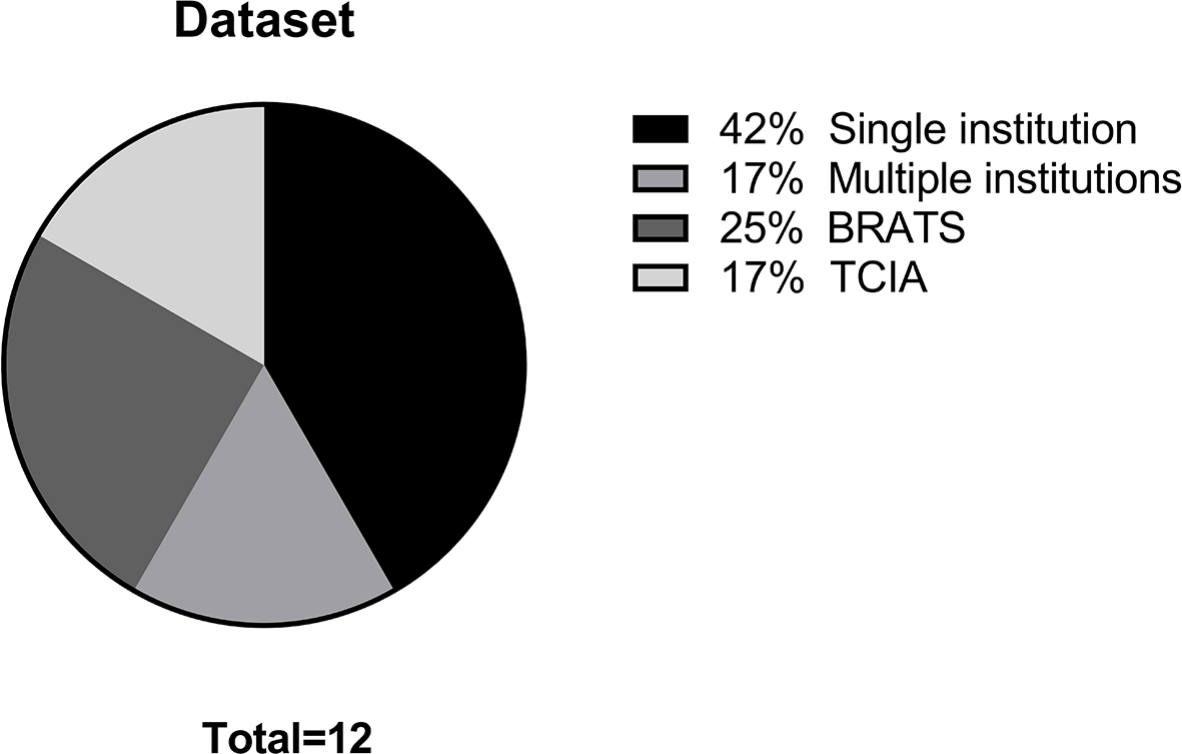
Distribution of datasets.

**Figure 4:**
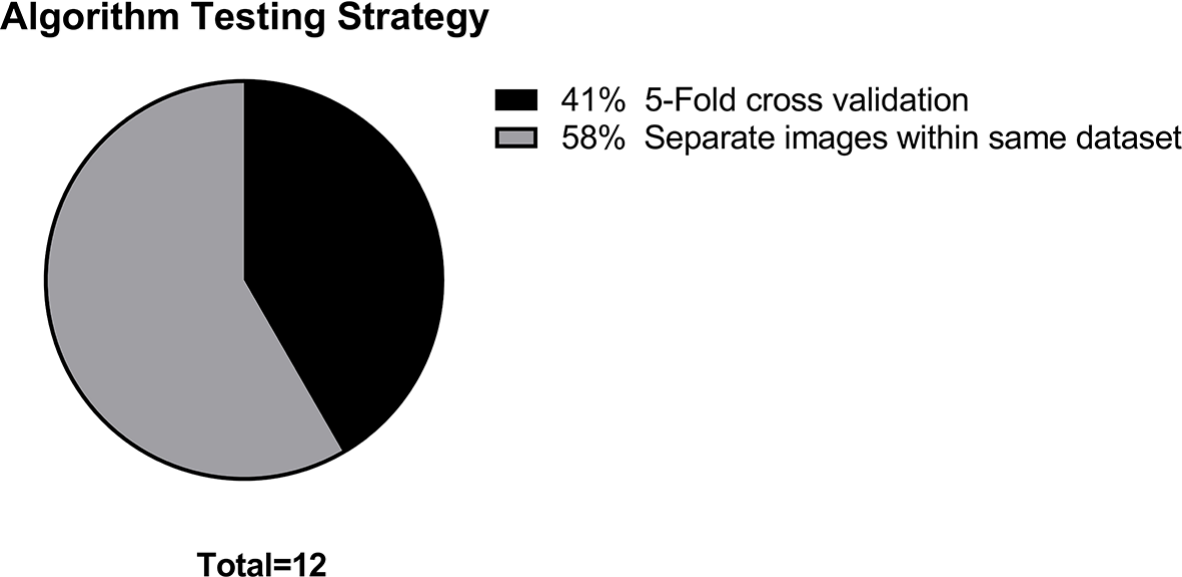
Distribution of machine learning algorithm testing strategies.

The main data points extracted from the 12 articles are summarized in **Table 1**. The distribution of the objective of the articles is depicted in **Figure 2**, the distribution of datasets utilized is depicted in **Figure 3**, and algorithm testing strategies are depicted in **Figure 4**. Seven articles investigated the differentiation of normal from abnormal images in datasets which include gliomas, and five articles investigated the differentiation of glioma images from non-glioma or normal images. The most frequent dataset used was a single institution dataset (5 articles, of which 4 used the Harvard Medical School dataset), followed by the Multimodal Brain Tumor Image Segmentation Benchmark (BRATS; 3 articles), multicenter datasets (2 articles), and The Cancer Imaging Archive (TCIA; 2 articles). BRATS (19–21) and TCIA (22) are publicly available databases of annotated MR images of gliomas. The ground truth in the BRATS and TCIA datasets is defined by pathology. Additionally, there was pathologic ground truth in the single institution dataset used by Dube et al. In the Harvard Medical School (23) dataset used by four studies, the method of ground truth establishment is unknown. Additionally, in the two studies using other multicenter datasets, the method to establish ground truth is unknown for at least part of the data.

**Table 1:** Summary of articles (n=12).

Author	Year of Publication	Purpose	Dataset	Ground Truth	Number of Patients	Training Strategy	Validation Strategy	Testing Strategy	MRI Sequences
Al-Saffar et al. (7)	2020	Glioma *vs*. Normal	TCIA 2013	Pathology	130	5-Fold Cross Validation	5-Fold Cross Validation	Separate images within same dataset	FLAIR
Kaur et al. (10)	2020	Normal *vs*. Abnormal	Multicenter	Unknown	717	Separate images within same dataset	None	Separate images within same dataset	T1, T1c, T2, FLAIR
Kharrat et al. (11)	2020	Glioma *vs*. Normal	BRATS 2013 and 2015	Pathology	304	5-Fold Cross Validation	None	5-Fold Cross Validation	T1, T1c, T2, FLAIR
Reddy et al. (12)	2020	Normal *vs*. Abnormal	Harvard Medical School	Unknown	Not specified (298 images)	5-Fold Cross Validation	None	5-Fold Cross Validation	T2
Samikannu et al. (14)	2020	Glioma *vs*. Normal	BRATS 2015	Pathology	176	Separate images within same dataset	None	Separate images within same dataset	Not specified
Ural et al. (16)	2020	Normal *vs*. Abnormal	Multicenter	Unknown	300	Separate images within same dataset	None	Separate images within same dataset	T1, T1c, T2, FLAIR, DWI
Kale et al. (9)	2019	Normal *vs*. Abnormal	Harvard Medical School	Unknown	Not specified (400 images)	5-Fold Cross Validation	None	5-Fold Cross Validation	T2
Rudie et al. (13)	2019	Glioma *vs*. Non-glioma	BRATS 2018	Pathology	351	10-Fold Cross Validation	10-Fold Cross Validation	Separate images within same dataset	T1, T1c, T2, FLAIR
Talo et al. (15)	2019	Normal *vs* abnormal	Harvard Medical School	Unknown	42	5-Fold Cross Validation	None	5-Fold Cross Validation	T2
Wong et al. (17)	2018	Glioma *vs*. Normal	TCIA 2017	Pathology	280	Separate images within same dataset	None	Separate images within same dataset	T1c
Zhang et al. (18)	2013	Normal *vs*. Abnormal	Harvard Medical School	Unknown	Not specified (90 images)	5-Fold Cross Validation	None	5-Fold Cross Validation	T2
Dube et al. (8)	2006	Normal *vs*. Abnormal	UCLA Brain Tumor Database	Pathology	60	Separate images within same dataset	None	Separate images within same dataset	T2

**Figure 5:**
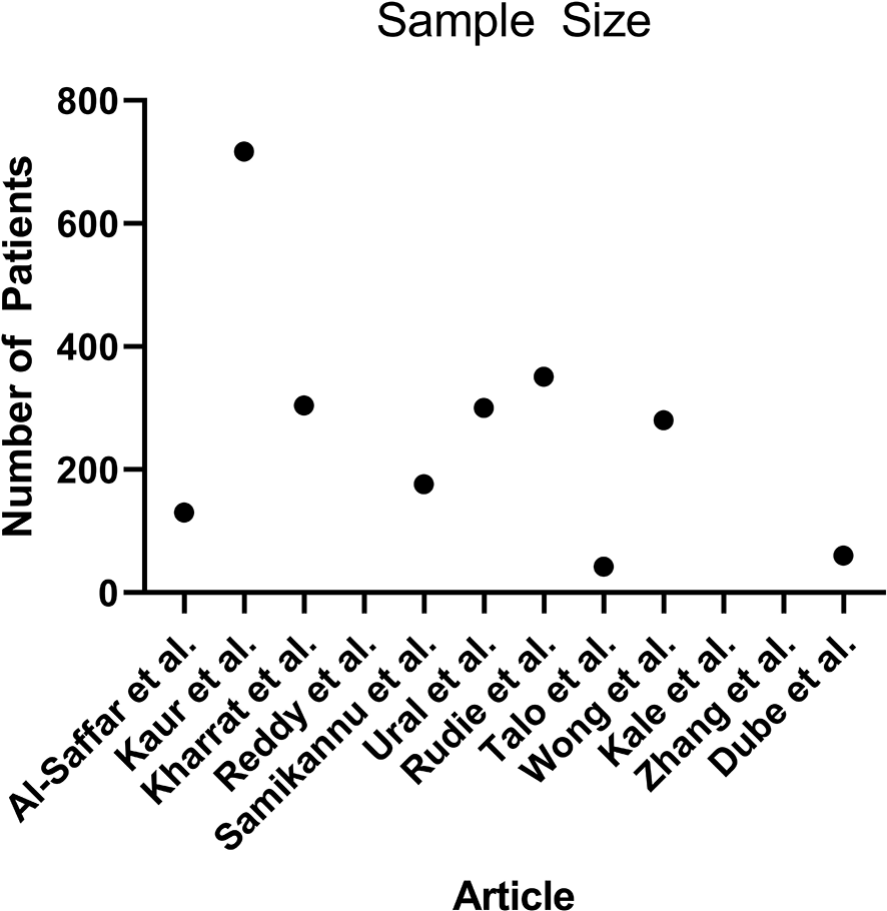
Scatterplot demonstrating the number of patients used in each article (n = 9, 3 articles did not report the number of patients).

Algorithm training and testing strategies consisted of five-fold cross validation (5 articles), and use of exclusive sets of images within the same dataset for training and for testing (7 articles). The range of sample sizes is shown in **Figure 5**. The median sample size was 280 patients (reported in 9 articles, range 42 to 717). The three articles not reporting the number of patients did report the number of images, with a median of 298 images (range 90 to 400). The sequences of magnetic resonance images used in each study was variable, consisting of some combination of T1-weighted, T2-weighted, contrast enhanced T1-weigthed, T2 fluid attenuated inversion recovery (FLAIR), and diffusion weighted (DWI) images.

A description of the machine learning algorithms is presented in **Table 2**. The most common algorithm was a neural network used in 10 articles, while two articles used support vector machine algorithms. A wide variety of neural networks were used, including five articles which developed novel algorithms. The quantitative results are demonstrated in **Table 3**, which summarizes the testing performance of each algorithm, and includes accuracy, sensitivity, specificity, AUC, and Dice coefficient. When multiple algorithms were evaluated in a study, the best performing algorithm was reported. The most commonly reported metric was accuracy, which ranged from 0.75 to 1.00 (median 0.96, 10 articles). When segmentation was investigated, the Dice coefficient was reported, which ranged from 0.92 to 0.98 (2 articles). A random effects meta-analysis was attempted, however could not be performed due to the lack of available data (24). The AUC was reported in only one of 12 articles and therefore not suitable for meta-analysis. Furthermore, for algorithm accuracy the standard deviation or confidence interval was only reported in three articles and therefore also not sufficient to perform an unbiased and generalizable meta-analysis (25).

**Table 2:** Summary of machine learning algorithms (n=12).

Author	Year of Publication	Purpose	Machine Learning Algorithm	Neural Network Type
Al-Saffar et al. (7)	2020	Glioma *vs*. Normal	Neural network	Novel (residual neural network)
Kaur et al. (10)	2020	Normal *vs*. Abnormal	Neural network	AlexNet, GoogleNet, ResNet50, ResNet101, VGG, VGG-19, InceptionV3, and InceptionResNetV2
Kharrat et al. (11)	2020	Glioma *vs*. Normal	Neural network	Novel (3D neural network)
Reddy et al. (12)	2020	Normal *vs*. Abnormal	Neural network	Novel (extreme learning machine)
Samikannu et al. (14)	2020	Glioma *vs*. Normal	Neural network	Novel (convolutional neural network)
Ural et al. (16)	2020	Normal *vs*. Abnormal	Neural network	Modified AlexNet and VGG
Kale et al. (9)	2019	Normal *vs*. Abnormal	Neural network	Novel (back propagation neural network)
Rudie et al. (13)	2019	Glioma *vs*. Non-glioma	Neural network	3D U-Net
Talo et al. (15)	2019	Normal *vs* abnormal	Neural network	ResNet34
Wong et al. (17)	2018	Glioma *vs*. Normal	Neural network	Modified VGG
Zhang et al. (18)	2013	Normal *vs*. Abnormal	Support vector machine	N/A
Dube et al. (8)	2006	Normal *vs*. Abnormal	Support vector machine	N/A

N/A, Not applicable.

**Table 3:** Summary of algorithm testing performance (n=12).

Author	Year of Publication	Purpose	Machine Learning Algorithm	Accuracy (Standard Deviation)	Sensitivity	Specificity	AUC	Dice coefficient
Al-Saffar et al. (7)	2020	Glioma *vs*. Normal	Novel (residual neural network)	0.9491 (NR)	0.9689	0.9637	NR	NR
Kaur et al. (10)	2020	Normal *vs*. Abnormal	AlexNet	1 (0)	1	1	1	NR
Kharrat et al. (11)	2020	Glioma *vs*. Normal	Novel (3D neural network)	NR	NR	NR	NR	0.98
Reddy et al. (12)	2020	Normal *vs*. Abnormal	Novel (extreme learning machine)	0.94 (0.23)	0.95	0.95	NR	NR
Samikannu et al. (14)	2020	Glioma *vs*. Normal	Novel (convolutional neural network)	0.991 (NR)	0.971	0.987	NR	NR
Ural et al. (16)	2020	Normal *vs*. Abnormal	Modified AlexNet and VGG	0.927 (NR)	0.968	0.98	NR	NR
Kale et al. (9)	2019	Normal *vs*. Abnormal	Novel (back propagation neural network)	1.0 (0.0002)	NR	NR	NR	NR
Rudie et al. (13)	2019	Glioma *vs*. Non-glioma	3D U-Net	NR	NR	NR	NR	0.92
Talo et al. (15)	2019	Normal *vs* abnormal	ResNet34	0.9787 (NR)	NR	NR	NR	NR
Wong et al. (17)	2018	Glioma *vs*. Normal	Modified VGG	0.82 (NR)	NR	NR	NR	NR
Zhang et al. (18)	2013	Normal *vs*. Abnormal	Support vector machine	0.9778 (NR)	0.9812	0.92	NR	NR
Dube et al. (8)	2006	Normal *vs*. Abnormal	Support vector machine	0.75 (NR)	NR	NR	NR	NR

NR, Not reported.

**Figure 6:**
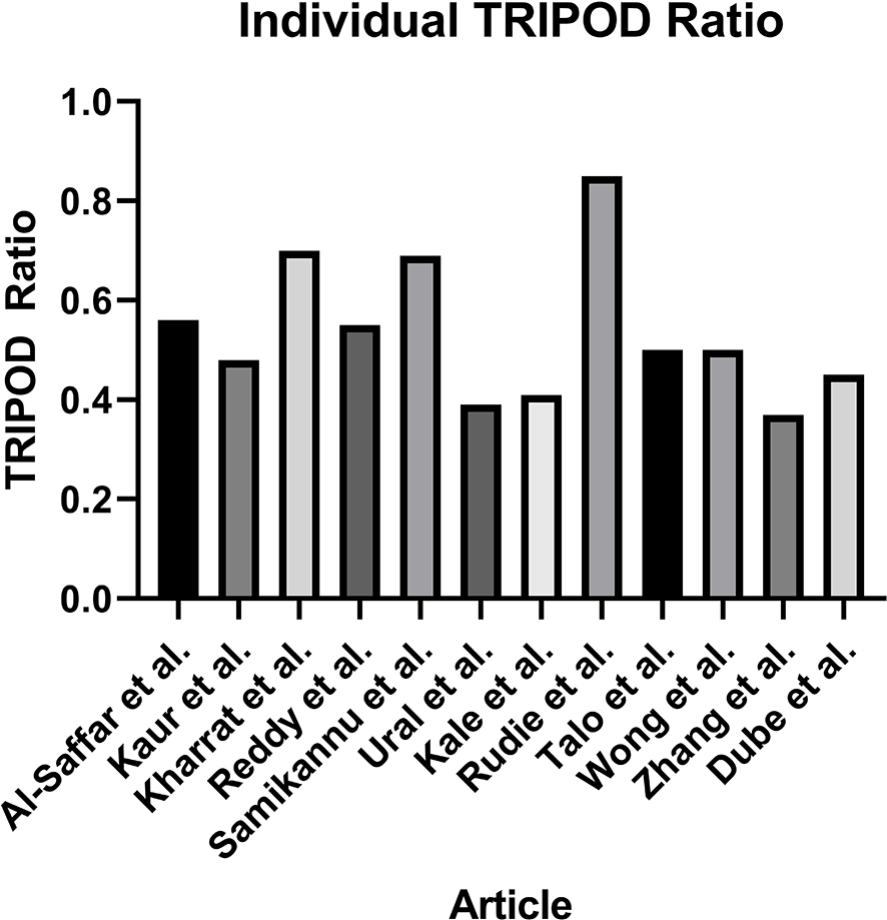
Individual TRIPOD Ratio, calculated for each article as the ratio of the TRIOPD score to the maximum possible score.

**Figure 7:**
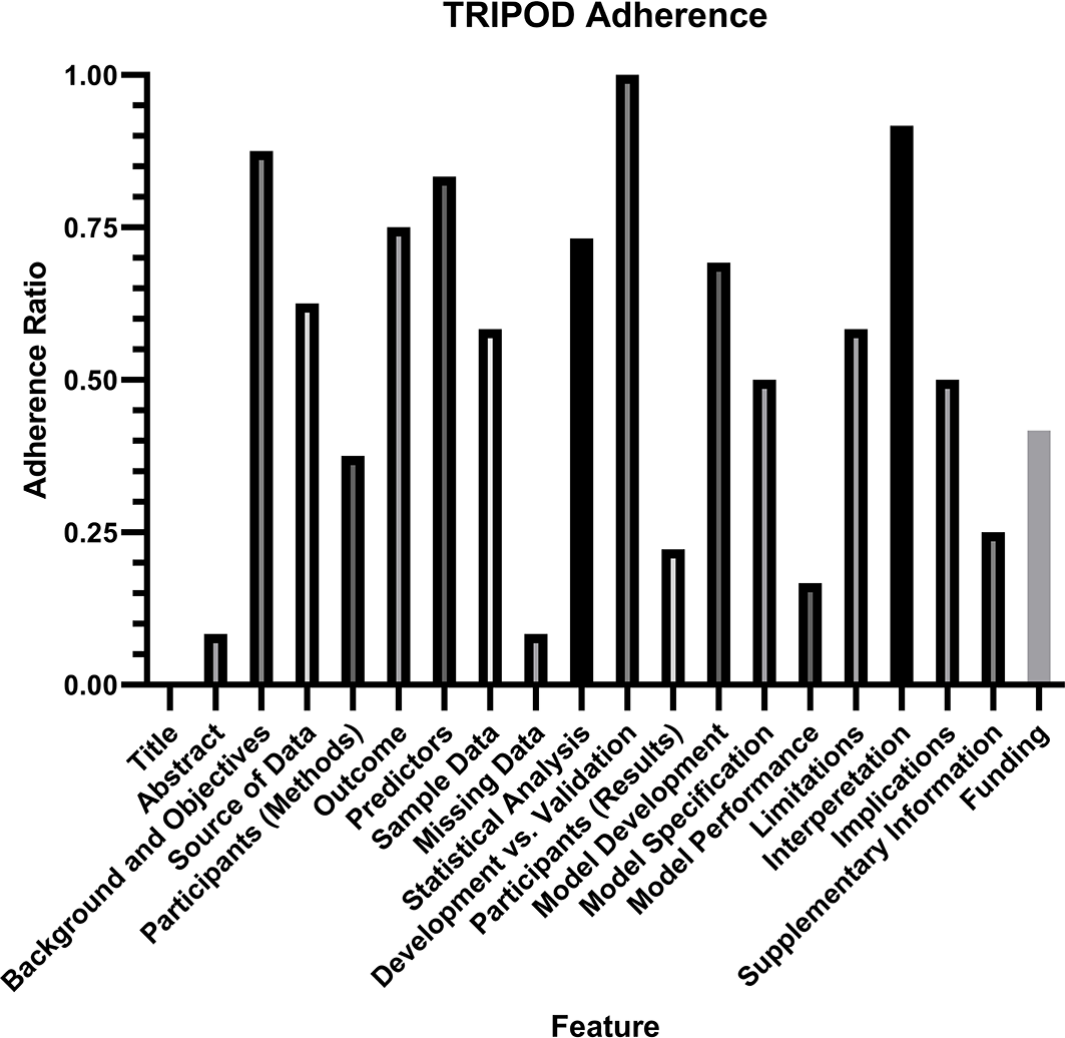
TRIPOD Adherence Ratio, calculated for each feature as the ratio of the total points scored to the total possible points for that feature. Notably, two features (risk groups and model updating) were not assessed in any article and therefore not included in the analysis.

Assessment of the quality of reporting using TRIPOD criteria yielded a mean individual TRIPOD ratio of 0.50 (standard deviation 0.14, range 0.37 to 0.85). Individual TRIPOD scores are depicted in **Figure 6** and feature TRIPOD adherence scores are depicted in **Figure 7**. Due to the inherent nature of the articles, no study created risk groups or discussed model updating. Both subitems of model specification were also not fully discussed in any article. In addition, both subitems of model development were fully included in only two articles. The maximum possible points for an individual article ranged from 26 to 29 when accounting for non-applicable features (the theoretical maximum points with all features included would be 37). Of the eligible features, the poorest adherence was seen with the title (0 adherent articles), abstract (1 adherent article), missing data (1 adherent article), results - participants (0 adherent articles) and model performance (2 adherent articles).

Additional linear regression analysis was performed to identify any predictor of algorithm accuracy. A comparison of algorithm accuracy and the sample size is demonstrated in **Supplementary Figure S2**, and shows no significant relationship, with an R^2^ of 0.1204 (P = 0.45). A comparison of algorithm accuracy and the individual TRIPOD ratio is demonstrated in **Supplementary Figure S3**, and shows no significant relationship, with an R^2^ of 0.01578 (P = 0.73).

## Discussion

A systematic review of the literature identified 12 studies which investigate the use of machine learning to identify gliomas in datasets which include non-glioma images. This scenario most closely simulates routine clinical practice, where gliomas are intermixed with normal examinations and non-oncologic pathologies. Moreover, these algorithms may have the potential to support a screening program for gliomas in the future. The studies were all published between 2006 and 2020, with nine published from 2019 to 2020, reflecting the increasing popularity of machine learning research in recent years. The five studies using BRATS or TCIA datasets included only normal images or glioma images. These datasets are more generalizable to clinical practice than those containing only glioma images, however still lack other routinely encountered pathologies. The remaining seven studies utilizing other single institution or multicenter datasets included a mix of normal, glioma, and other pathologic images. The other pathologies included stroke, Alzheimer’s disease, multiple sclerosis, and meningioma, among others. This data is more representative of routine clinical practice, however still comes with limitations. There are a wide variety of healthcare settings, such as a tertiary or academic medical center, small hospital, or outpatient practice, each with different patient populations and pathologies. Additionally, datasets from different locations around the world will demonstrate different heterogeneity based on regional variations.

There are major limitations with the algorithm training and testing strategies. The description of algorithm training, validation, and testing strategies is heterogenous across studies. Often in machine learning research, validation and testing are used interchangeably, however this leads to confusion in the evaluation of algorithm performance. Validation should be reserved for the description of algorithm finetuning using data separate from the training data. Testing should be used to describe the unbiased evaluation of an algorithm using data separate from the training and validation sets. Each study reported training and testing data, however many studies used the term validation for what should actually be described as testing. Only two studies performed a true validation in addition to training and testing, Al-Saffar et al. used 5-fold cross validation for training and validation followed by a separate set of images within the same dataset for testing, and Rudie et al. used 10-fold cross validation for training and validation followed by a separate set of images within the same dataset for testing. None of the 12 studies tested their algorithms on external data. This poses a major limitation to the generalizability of these algorithms. In the United States, this also hinders the ability for approval by the Food and Drug Administration, which recommends algorithms be tested on external datasets.

Overall, there appears to be limited availability of high-quality data to train these machine learning algorithms. The number of patients in the datasets was low, with no study reaching 1,000 patients, and one study dropping as low as 42 patients. As a result of low sample sizes, the k-fold cross validation technique was commonly used for algorithm training, and five studies even used k-fold cross validation to test their algorithms. This technique is optimal for providing more data with a small sample size, but comes with the drawback of increased overtraining and decreased generalizability when applying the algorithm to an outside dataset. Additionally, nine studies used the same three datasets: BRATS, TCIA, and Harvard Medical School. Only two studies used datasets compiled from multiple institutions. This highlights a need to develop larger and more clinically applicable datasets to perform more robust machine learning research. Moreover, it will be critical to develop datasets that closely represent the mix of pathology encountered in each individual hospital, because this will vary between different institutions and practice settings. This will potentially fabricate the need for hospital specific dataset creation for the translation of algorithms.

Risk of bias analysis using TRIPOD criteria revealed that the quality of reporting was insufficient to draw any conclusion about algorithm generalizability. On average, there was adherence to only half of the reporting standards, with a large variation between studies. The poorest adherence was noted with the title and abstract, the method for handling missing data, the description of study participants within the results section, and the reporting of model performance. Specifically for model performance, the confidence interval of the discrimination measure was reported in only two studies. It is important to note that the TRIOPD criteria were primary developed for studies that used conventional multivariate regression prediction models rather than machine learning models, and TRIPOD-AI criteria are currently in development to specifically address the reporting of artificial intelligence and machine learning models (26). Poor quality of reporting also limited the ability to perform a meta-analysis, as AUC was reported in only one study, and the standard deviation for accuracy was reported in only three studies. Overall, the current analysis demonstrates that a substantial portion of information needed for translating algorithms to clinical practice is not available.

## Conclusion

Systematic review of the literature identified machine learning algorithms which can identify gliomas in datasets containing non-glioma images, which are the most suitable algorithms for integration into general clinical workflow. Such algorithms may also serve as the basis for a potential brain tumor screening program. Severe limitations hindering the application of these algorithms to clinical practice were identified, including limited datasets, the lack of generalizable algorithm training and testing strategies, and poor quality of reporting. There is a need to develop more robust and heterogeneous datasets, which can be applied to individual clinical practice settings. Future studies would benefit from using external datasets for algorithm testing as well as placing increased attention on quality of reporting standards.

## Data Availability Statement

The original contributions presented in the study are included in the article/**Supplementary Material**. Further inquiries can be directed to the corresponding author.

## Author Contributions

All authors listed have made a substantial, direct, and intellectual contribution to the work and approved it for publication.

## Funding

This publication was made possible by KL2 TR001862 (MA) from the National Center for Advancing Translational Science (NCATS), components of the National Institutes of Health (NIH), and NIH roadmap for Medical Research. This publication also received support from the American Society of Neuroradiology Fellow Award 2018 (MA).

## Author Disclaimer

Its contents are solely the responsibility of the authors and do not necessarily represent the official view of NIH.

## Conflict of Interest

The authors declare that the research was conducted in the absence of any commercial or financial relationships that could be construed as a potential conflict of interest.

## Publisher’s Note

All claims expressed in this article are solely those of the authors and do not necessarily represent those of their affiliated organizations, or those of the publisher, the editors and the reviewers. Any product that may be evaluated in this article, or claim that may be made by its manufacturer, is not guaranteed or endorsed by the publisher.

## Supplementary Material

Supplementary Figure 1 Search strategy and syntax. A total of four databases were searched: Embase, Ovid MEDLINE(R), Cochrane CENTRAL (trials), and Web of Science.

Supplementary Figure 2 Linear regression analysis demonstrates no significant relationship between algorithm accuracy and sample size (R^2^ of 0.1204, P = 0.45).

Supplementary Figure 3 Linear regression analysis demonstrates no significant relationship between algorithm accuracy and individual TRIOPD ratio (R^2^ of 0.01578, P = 0.73).
