# Supplementary figures and images for: Trends in Development of Novel Machine Learning Methods for the Identification of Gliomas in Datasets That Include Non-Glioma Images: A Systematic Review
Source: Front Oncol. 2021 Dec 23;11:788819. doi: 10.3389/fonc.2021.788819 (PMC8733688; doi:10.3389/fonc.2021.788819)

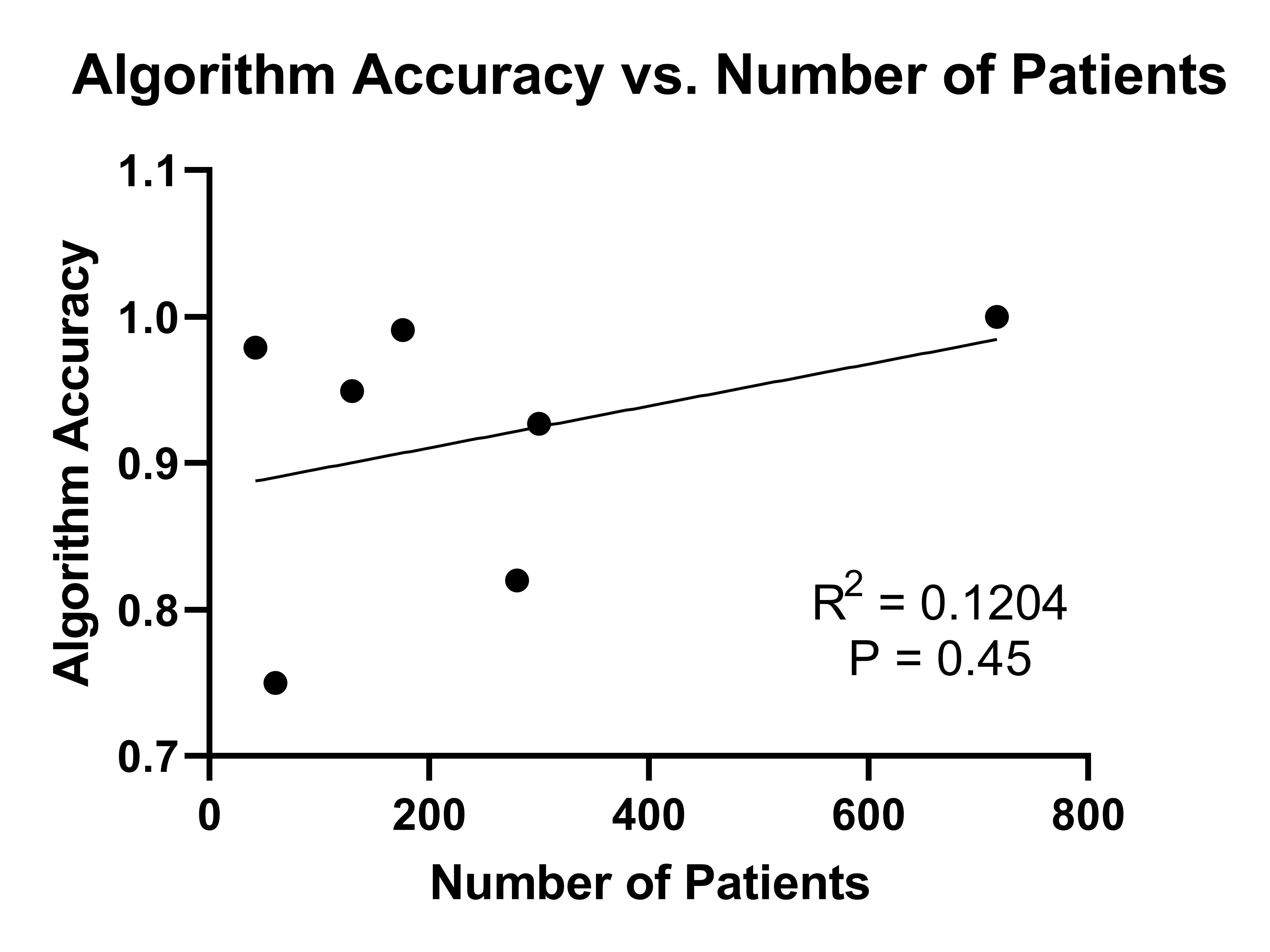

Supplement: Supplementary Figure 1 — Search strategy and syntax. A total of four databases were searched: Embase, Ovid MEDLINE(R), Cochrane CENTRAL (trials), and Web of Science. [file Image_1.jpeg]

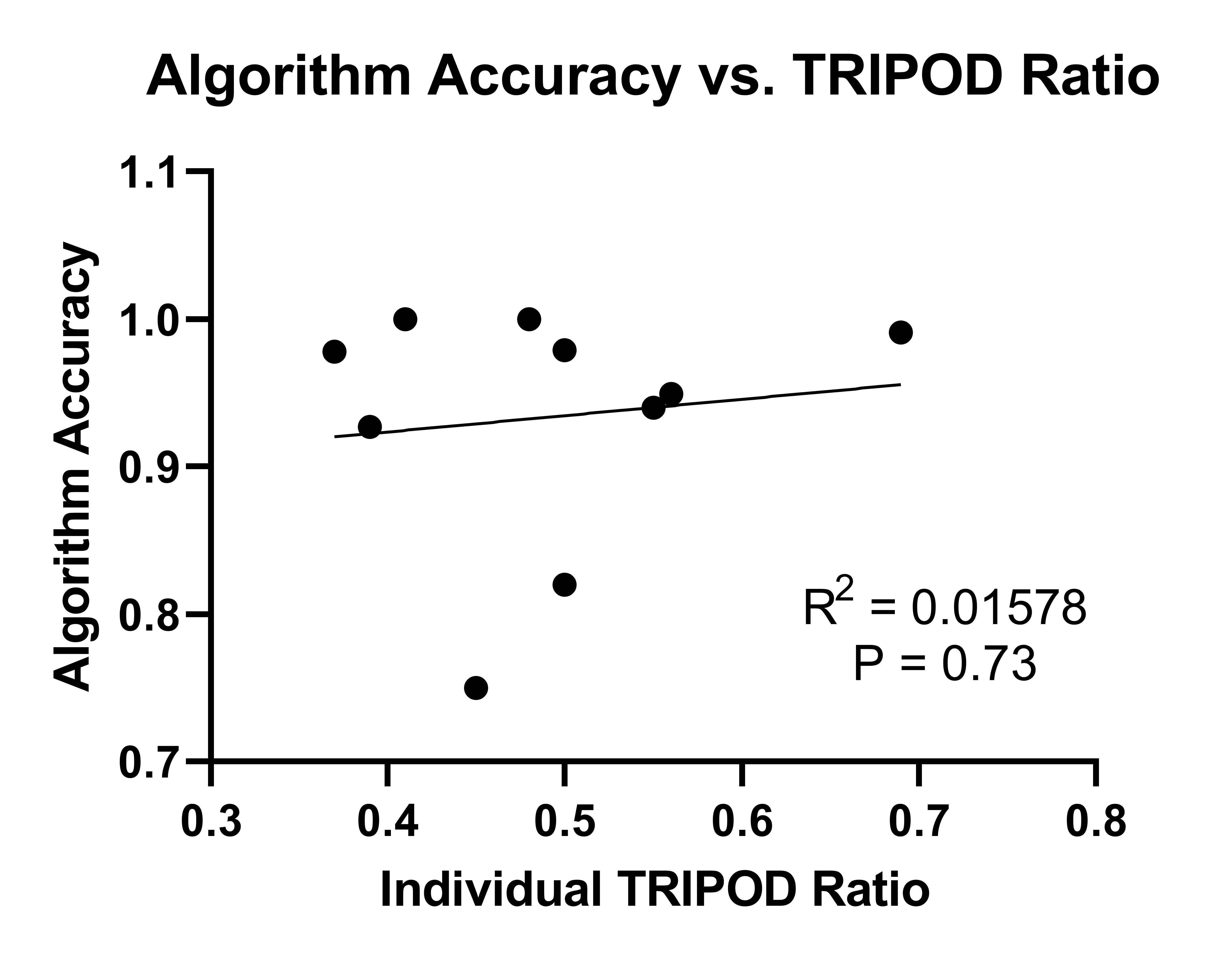

Supplement: Supplementary Figure 2 — Linear regression analysis demonstrates no significant relationship between algorithm accuracy and sample size (R2 of 0.1204, P = 0.45). [file Image_2.jpeg]
