# Supplementary material for: Trends in Development of Novel Machine Learning Methods for the Identification of Gliomas in Datasets That Include Non-Glioma Images: A Systematic Review
Source: Front Oncol. 2021 Dec 23;11:788819. doi: 10.3389/fonc.2021.788819 (PMC8733688; doi:10.3389/fonc.2021.788819)
Supplement: Supplementary Figure 3 — Linear regression analysis demonstrates no significant relationship between algorithm accuracy and individual TRIOPD ratio (R2 of 0.01578, P = 0.73). [file DataSheet_1.docx]

**Supplementary Figure S1. Search strategy and syntax. A total of four databases were searched: Embase, Ovid MEDLINE(R), Cochrane CENTRAL (trials), and Web of Science.**

**Embase** <1974 to 2021 January 29>

1 exp Artificial Intelligence/ 45007

2 machine learning/ 37007

3 deep learning/ 12393

4 ((artificial* or machine* or deep*) adj3 (intelligence or learning)).tw,kw. 73708

5 AI.ti,ab. 39008

6 exp computer assisted diagnosis/ 1169775

7 computer* assist* diagnosis.tw,kw. 937

8 radiomics/ 1903

9 radiomic*.tw,kw. 4867

10 or/1-9 1305625

11 exp nuclear magnetic resonance imaging/ 1001848

12 (Magnetic Resonance Imag* or MR-Imag* or MR Imag or MRI* or NMR).tw,kw. 882389

13 11 or 12 1278307

14 exp glioma/ 139715

15 glioma*.tw,kw. 84577

16 (glial adj2 (tumor* or tumour*)).tw,kw. 3616

17 (glioblastoma* or astrocytoma* or astrocytic glioma* or astroglioma).tw,kw. 77347

18 or/14-17 165100

19 10 and 13 and 18 9560

20 limit 19 to yr="2020 - 2022" 771

**Ovid MEDLINE(R**) ALL <1946 to January 29, 2021>

1 exp Artificial Intelligence/ 106412

2 ((artificial* or machine* or deep*) adj3 (intelligence or learning)).tw,kw. 55350

3 AI.ti,ab. 28603

4 exp Image Interpretation, Computer-Assisted/ 551508

5 computer* assist* diagnosis.tw,kw. 626

6 radiomic*.tw,kw. 3204

7 or/1-6 706556

8 exp Magnetic Resonance Imaging/ 465045

9 (Magnetic Resonance Imag* or MR-Imag* or MR Imag or MRI*).tw,kw. 435821

10 8 or 9 625065

11 exp Glioma/ 85314

12 glioma*.tw,kw. 60258

13 (glial adj2 (tumor or tumour)).tw,kw. 831

14 (glioblastoma* or astrocytoma* or astrocytic glioma* or astroglioma).tw,kw. 51784

15 or/11-14 115828

16 7 and 10 and 15 4493

17 limit 16 to yr="2020 - 2021" 260

**Cochrane CENTRAL (trials)**

ID Search Hits

#1 MeSH descriptor: [Artificial Intelligence] explode all trees 1040

#2 (artificial* OR machine* OR deep*) AND (intelligence OR learning) 3131

#3 AI 7937

#4 MeSH descriptor: [Image Processing, Computer-Assisted] explode all trees 3582

#5 computer* assist* diagnosis 6489

#6 radiomic* 210

#7 #1 OR #2 OR #3 OR #4 OR #5 OR #6 20843

#8 MeSH descriptor: [D008279] explode all trees 0

#9 Magnetic Resonance Imag* OR MR-Imag* OR MR Imag OR MRI* OR NMR 36332

#10 #8 OR #9 36332

#11 MeSH descriptor: [Glioma] explode all trees 1197

#12 glioma* 1792

#13 (glial AND (tumor OR tumour)) 70

#14 glioblastoma* OR astrocytoma* OR astrocytic glioma* OR astroglioma 2432

#15 #11 OR #12 OR #13 OR #14 3580

#16 #7 AND #10 AND #15 with Publication Year from 2020 to 2021, in Trials 2

**Web of Science**

# 13

235

#12

Indexes=SCI-EXPANDED, SSCI, A&HCI, CPCI-S, CPCI-SSH, BKCI-S, BKCI-SSH, ESCI, CCR-EXPANDED, IC Timespan=2020-2021

# 12

711

#11 AND #7 AND #6

Indexes=SCI-EXPANDED, SSCI, A&HCI, CPCI-S, CPCI-SSH, BKCI-S, BKCI-SSH, ESCI, CCR-EXPANDED, IC Timespan=All years

# 11

132,043

#10 OR #9 OR #8

Indexes=SCI-EXPANDED, SSCI, A&HCI, CPCI-S, CPCI-SSH, BKCI-S, BKCI-SSH, ESCI, CCR-EXPANDED, IC Timespan=All years

# 10

75,253

TS=(glioblastoma* or astrocytoma* or astrocytic glioma* or astroglioma)

Indexes=SCI-EXPANDED, SSCI, A&HCI, CPCI-S, CPCI-SSH, BKCI-S, BKCI-SSH, ESCI, CCR-EXPANDED, IC Timespan=All years

# 9

2,783

TS=(glial NEAR/2 (tumor* or tumour*))

Indexes=SCI-EXPANDED, SSCI, A&HCI, CPCI-S, CPCI-SSH, BKCI-S, BKCI-SSH, ESCI, CCR-EXPANDED, IC Timespan=All years

# 8

91,055

TS=(glioma*)

Indexes=SCI-EXPANDED, SSCI, A&HCI, CPCI-S, CPCI-SSH, BKCI-S, BKCI-SSH, ESCI, CCR-EXPANDED, IC Timespan=All years

# 7

1,046,719

TS=(Magnetic Resonance Imag* or MR-Imag* or MR Imag or MRI* or NMR)

Indexes=SCI-EXPANDED, SSCI, A&HCI, CPCI-S, CPCI-SSH, BKCI-S, BKCI-SSH, ESCI, CCR-EXPANDED, IC Timespan=All years

# 6

331,970

#5 OR #4 OR #3 OR #2 OR #1

Indexes=SCI-EXPANDED, SSCI, A&HCI, CPCI-S, CPCI-SSH, BKCI-S, BKCI-SSH, ESCI, CCR-EXPANDED, IC Timespan=All years

# 5

4,619

TS=(radiomic*)

Indexes=SCI-EXPANDED, SSCI, A&HCI, CPCI-S, CPCI-SSH, BKCI-S, BKCI-SSH, ESCI, CCR-EXPANDED, IC

# 4

6,845

TS=(computer* assist* diagnosis)

Indexes=SCI-EXPANDED, SSCI, A&HCI, CPCI-S, CPCI-SSH, BKCI-S, BKCI-SSH, ESCI, CCR-EXPANDED, IC Timespan=All years

# 3

51,251

AB=(AI)

Indexes=SCI-EXPANDED, SSCI, A&HCI, CPCI-S, CPCI-SSH, BKCI-S, BKCI-SSH, ESCI, CCR-EXPANDED, IC Timespan=All years

# 2

11,596

TI=(AI)

Indexes=SCI-EXPANDED, SSCI, A&HCI, CPCI-S, CPCI-SSH, BKCI-S, BKCI-SSH, ESCI, CCR-EXPANDED, IC Timespan=All years

# 1

276,118

TS=((artificial* or machine* or deep*) NEAR/3 (intelligence or learning))

Indexes=SCI-EXPANDED, SSCI, A&HCI, CPCI-S, CPCI-SSH, BKCI-S, BKCI-SSH, ESCI, CCR-EXPANDED, IC Timespan=All years
